# Prolonged Persistence of Chimeric Antigen Receptor (CAR) T Cell in Adoptive Cancer Immunotherapy: Challenges and Ways Forward

**DOI:** 10.3389/fimmu.2020.00702

**Published:** 2020-04-22

**Authors:** Leila Jafarzadeh, Elham Masoumi, Keyvan Fallah-Mehrjardi, Hamid Reza Mirzaei, Jamshid Hadjati

**Affiliations:** ^1^Department of Medical Immunology, School of Medicine, Tehran University of Medical Sciences, Tehran, Iran; ^2^Department of Medical Immunology, School of Medicine, Ilam University of Medical Sciences, Ilam, Iran

**Keywords:** chimeric antigen receptor, T cells, persistence, tumor microenvironment, cancer immunotherapy

## Abstract

CAR T cell qualities, such as persistence and functionality play important roles in determining the outcome of cancer immunotherapy. In spite of full functionality, it has been shown that poor persistence of CAR T cells can limit an effective antitumor immune response. Here, we outline specific strategies that can be employed to overcome intrinsic and extrinsic barriers to CAR T cell persistence. We also offer our viewpoint on how growing use of CAR T cells in various cancers may require modifications in the intrinsic and extrinsic survival signals of CAR T cells. We anticipate these amendments will additionally provide the rationales for generation of more persistent, and thereby, more effective CAR T cell treatments. CAR T cell qualities, such as persistence and functionality play important roles in determining the outcome of cancer immunotherapy. In spite of full functionality, it has been shown that poor persistence of CAR T cells can limit an effective antitumor immune response. Here, we outline specific strategies that can be employed to overcome intrinsic and extrinsic barriers to CAR T cell persistence. We also offer our viewpoint on how growing use of CAR T cells in various cancers may require modifications in the intrinsic and extrinsic survival signals of CAR T cells. We anticipate these amendments will additionally provide the rationales for generation of more persistent, and thereby, more effective CAR T cell treatments.

## Introduction

Although chimeric antigen receptor (CAR) T cell therapy has made remarkable strides in the treatment of patients with difficult to treat cancers, strategies must be developed to benefit great numbers of individuals with relapsed and refractory tumors. It is evident that several barriers need to be overcome and/or still identified before this type of therapy becomes a widely accepted standard treatment protocol for different cancers. Among the barriers, poor persistence of infused cells is a critical challenge in successful cancer therapy. It has been well-recognized that poor persistence of infused CAR T cells is inversely correlated with durable clinical remissions in patients with cancers. Indeed, poor persistence hinders the long-term effector functions of infused cells *in vivo* and potentially hampers the long-term therapeutic impacts of CAR T cell therapy. Several factors can influence the persistence of adoptively transferred T cells. Here, we will discuss multiple strategies to enhance CAR T cell persistence and antitumor activity including optimized *ex vivo* T cell culture conditions, pre-treatment with specific conditioning regimens and pharmacological inhibitors, manipulations of genes involved in T cell survival (e.g., anti-apoptotic and proapoptotic genes and cytokines), modification of different parts of CAR construct, redox regulation system, reversing T cell exhaustion, blunting host immune responses against the cellular infusion product, T cell selection procedures, and ectopic expression of genes regulating cell survival (e.g., TERT), aiming to improve the outcome of therapy.

### *Ex vivo* Cell Culture Conditions

It has been well-recognized that *ex vivo* culturing condition is one of the influential factors on the differentiation status and survival of CAR T cells. To obtain sufficient numbers of T cells for infusion, it is also required to culture and expand T cells *ex vivo*. Many of the currently used cell culture protocols are laborious and time-consuming which may restrict their clinical application (1). Furthermore, some of these protocols produce a final T cell product dominated by fully differentiated T cells with a predisposition toward activation-induced cell death (AICD), and characterized by skewed T cell repertoire, and poor *in vivo* persistence. *Ex vivo* cell culture as a pivotal process for cell therapy is compulsory for clinical applications of CAR T cells, and variables include medium formulation (i.e., basal media and supplements such as type of cytokines and their concentrations), culturing time, cell seeding density, activation protocols for isolated T cells from the blood and subculture protocols. Cytokines as medium supplements are likely the most critical factors for *ex vivo* culture of CAR T cells. As cytokines are crucial in improving the survival of CAR T cells, we describe various detailed cytokine recipes which are commonly used for *ex vivo* expansion of CAR T cells.

Common γ chain (γc) cytokines (such as IL-4, IL-2, IL-7, IL-21, and IL-15) play a key role in the differentiation, development and survival of different immune cells. In the cancer immunotherapy, γc cytokines have been utilized as monotherapies to stimulate endogenous antitumor immunity, or in combination with adoptive cell therapy to improve antitumor efficiency. IL-2 is a potent T cell growth cytokine that largely affects the characteristics and effectiveness of T cells. This cytokine is regularly supplemented in the CAR T cell culture media. IL-2 is also necessary for survival of T regulatory cells. Although Tregs, through IL-2 consumption, impair proliferation of conventional T cells (2), the higher concentrations of IL-2 can stimulate conventional T cells (3). To improve the persistence of T cells (after infusion) within the patient body, IL−2 has been used in many clinical trials (4–6). However, its administration has been associated with some toxicities (7, 8) and expansion of Tregs (9). These adverse effects made the administration of IL- 2 limited and with considerations. Nevertheless many studies have been trying to modify IL-2 concentration and/or timing of supplementation in the *ex vivo* cell culture media to increase survival of CAR T cells. There are limited studies describing the effect of cytokine supplementation (rather than IL-2) on *ex vivo* persistence of CAR T cells. The advantage of using IL-2 in the culture media of CAR T cells is clear. The common concentration of IL-2 which has been used in the CAR T cell studies is between 50 to 100 IU/ml. Besser et al., have shown that both timing of supplementation and concentration of IL-2 have profound effects on growth, cytotoxicity, cytokine release, and surface marker expression of tumor-infiltrating lymphocytes. They found that a combined protocol of starting with 10–120 IU/ml IL-2 during the first week, followed by increasing IL-2 concentration to 6000 IU/mL within the second week, results in the generation of T cells that expand well, maximally produce IFN-γ and are highly cytotoxic against tumor cells. However, in this study, T cell survival and abundance of different subpopulations of memory T cells were not examined (10). Kaaratinen et al., have assessed the effects of different doses of IL-2 (0–300 IU/mL) and expansion duration (10–20 days) on the phenotype of T cell products during *in vitro* expansion. Their results showed that production of CAR T cells in the absence of IL-2 yields the highest amount of early functionally potent memory T cells and provides a 10-fold cell expansion (11). They also exhibited that high dose of IL-2 can inversely decrease overall generation of T memory stem cells (Tscm) by both reducing central memory T cells (Tcm) and expanding effector T cells (11). Zhang et al., have examined short and long-term effect of IL-2 on CD19-CAR T cells. They reported that long-term culture of CAR T cells with IL-2 promotes their terminal differentiation however, their short-term culture with IL-2 increases the generation of memory CAR T cells which are desirable subsets in CAR T cell cancer therapy (12). The result of this study is desirable where IL-2 is used for short-term culture aiming not only to improve *ex vivo* survival of CAR T cells following reinfusion to patients but also to prevent some adverse effects related to IL-2 administration such as expansion of regulatory T cells.

Besides IL-2, IL-7, and IL-15 cytokines are commonly used for *ex vivo* expansion of T cells. IL-7 signaling endorses the homeostasis and survival of memory and naïve T cells through upregulation of Bcl-2 and the repression of proapoptotic markers (13). IL-15R signaling and IL-15 are also essential for the homeostasis and development of CD8+ T cells via the inhibition of AICD and upregulation of antiapoptotic mediators such as Mcl1 and Bcl-2 (14). Ghassemi et al., showed that *ex vivo* short-term culture of primary T cells by IL-15 and IL-7 improves *in vivo* persistence of T cells. Cytokines IL-7 and IL-15 reduce the differentiation of Tscm and Tcm into effector T cells and, thereby, preserving phenotype of T cells in early differentiation status (15). Quintarelli et al., have shown that supplementation of T cell culture medium with IL-7 and IL-15 was found to be synergic with the anti-GD2 CAR design in enhancing the antitumor activity of CAR T cells in a neuroblastoma mouse model (16). Their results showed that IL-7 and IL-15 supplementation can enhance CAR T cell persistence by reversion of CAR T cell exhaustion. Xu et al., have found that culturing CAR T cells with IL-7 and IL-15 *ex vivo* increases the frequency of CAR T cells with memory stem cell phenotype. These CAR T cells are also able to produce greater antitumor activity through increased resistance to cell death, following repetitive encounters with the antigen, while preserving their migration to secondary lymphoid organs (17). Based on these studies and the effect of IL-15 and IL-7 on persistence and differentiation of memory T cells, it is clear that these cytokines can help scientists make long-lived CAR T cells through cell culture medium supplementation. However, to achieve the best results and efficacy, more investigations are required to identify the best method, incubation time, dose and signaling domains incorporated into CAR transgene.

The novel class I cytokine IL-21 is a member of the common γ-chain receptor family. This cytokine is able to synergize with IL-7 and IL15. For instance, IL-21 and IL-15 additively endorse the expansion of CD8+ Tscm cells and enhance T cell longevity when infused into the host (18). IL-15 and/or high doses of IL-7 are well-recognized to be essential for survival of memory (CD44high) CD8+ T cells (19). To generate long-lived Tscm cells, Alvarez-Fernandez et al., have also developed a protocol for *ex vivo* generation of Tscm cells based on CD3/CD28 costimulation and the use of cytokines IL-7, IL-15 and/or IL-21 during the entire culture period. Their results showed that a brief anti-CD3/CD28 costimulation of naïve T cells, combined with IL-7 and IL-15, significantly increase the frequency of Tscm cells *ex vivo*. Besides, they also showed that addition of IL-21 to this condition further supports the enrichment and expansion of Tscm cells with an increase in the absolute numbers (20). Other studies also have shown that *ex vivo* supplementation with IL-21 supports development and expansion of less differentiated memory CAR T cells with superior antitumor activity (21, 22). Considering the evidence that *in vivo* expansion of CAR T cells is correlated with the frequency of less differentiated CAR T cells within the infused products (23), it seems that *ex vivo* supplementation of CAR T cells with IL-21 alone or in combination with other cytokines (such as IL-7 and IL-15) has potential roles in the development and maintenance of significant fractions of less differentiated CAR T cells with superior antitumor activities.

**Figure 1:**
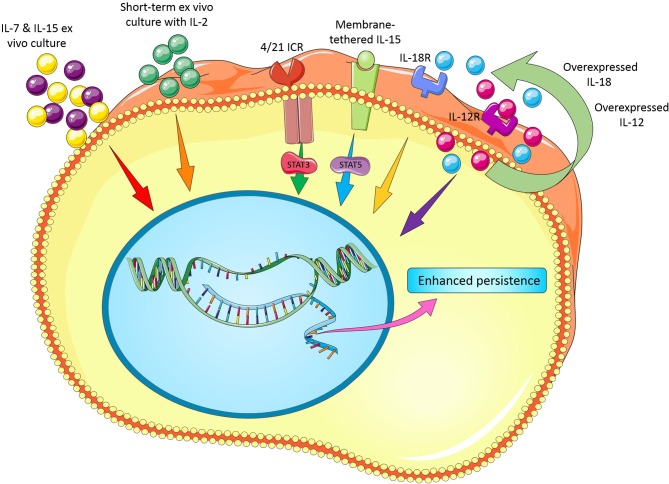
Novel modifications in cytokine recipe and their receptors to enhance CAR T cell persistence.

It is clear that the effect of cytokines on T cell survival *in vitro* is likely different as seen *in vivo* perhaps owing to the existence of some interactions between cytokines, other signaling molecules/pathways and microbiota. Furthermore, best concentration and incubation time for cytokine treatment, choosing the best cytokine cocktail with synergic effects, development of strategies for overexpression (e.g., viral vectors) or repression of some cytokines (e.g., TGF-β by siRNA, shRNA, CRISPR/Cas9, or antagonist drugs) are remained to be fully assessed. Figure 1 illustrates novel modifications in cytokine recipe to enhance CAR T cell persistence for adoptive cancer immunotherapy.

Human serum (HS) is another key component of medium formulation which is commonly used to expand CAR T cells. Since HS is expensive; has substantial inter-lot variations; needs regular screening and monitoring for the presence of potentially emerging infectious agents and the agents that detrimental for T cell expansion and survival; and also limited resources of HS if a very large-scale T cell therapy is aimed to be conducted (24). Thus, a T cell manufacturing process that is not dependent on HS may make CAR T cell therapy less expensive, more consistent, and available to more patients. To this end, Medvec et al., have developed a chemically defined HS- free cell culture medium (i.e., 1B2H medium) that vigorously expands all T cell subpopulations. Using a humanized murine model, they observed that T cells expanded in the HS-free cell culture medium could control tumor growth in a long-term time period, while T cells expanded in medium supplemented with HS could only control tumor growth in a short-term time period. These data obviously indicate that *ex vivo* expansion of T cells in the HS-free medium could improve both the functionality and durability of CAR T cells and this highlights the importance of *ex vivo* culture conditions in the survival and function of CAR T cells and should be taken into considerations in the manufacturing of these cells (25).

## Manipulating CAR T Cells to Express Cytokines and Their Receptors

Cytokines and their cognate receptors are key modulators of T cell maturation, activation, and proliferation. In the previous section, we focused on the importance of cytokine recipes in the *ex vivo* cell culture. In this section we specifically focus on how CAR-expressing T cells can be engineered to produce a wide range of cytokines (such as IL-4, IL-2, IL-7, IL-21, and IL-15) or their cognate receptors aiming to improve antitumor and persistence of these cells. So far, antitumor effect and survival advantage of these genetically engineered T cells has been investigated in a wide range of preclinical and clinical studies.

In a study, Adachi et al., engineered T cells to overexpress CAR, CCL19 and IL-7 transgenes (also called 7 × 19CAR T cells). *In vivo* data revealed that, compared to conventional CAR T cells, 7 × 19 CAR engineered T cells not only had superior antitumor activity but also could prolong the survival of tumor-bearing mice. Following treatment of tumor-bearing mice with engineered CAR T cells, both endogenous T cells and infused CAR T cells were able to induce memory antitumor immune responses. Their findings suggest that a suitable combination therapy of CAR T cells and immune-modulating factors like CCL19 and IL-7 can augment the antitumor potential of CAR T cells in coordination with activation of endogenous immune system and their memory formation (26).

In another study, Hoyos et al., reported that since *in vivo* expansion, survival, and antitumor activity of CD19 CAR T cells remain suboptimal even when the CAR construct contains a CD28 costimulatory molecule, they designed a novel construct that also included IL-15 gene and an inducible 5caspase-9-based suicide gene (also called iC9/CAR.19/IL-15). They found that compared to conventional CAR T cells, iC9/CAR.19/IL-15 T cells have greater antigen-dependent expansion, reduced cell death rate, and improved antitumor effects *in vivo* (27). Krenciute et al., have also shown that transgenic expression of IL-15 improves the persistence and proliferative capacity and, thereby, antitumor function of IL13Rα2-CAR T cells in a preclinical glioma model (28). In another study, Hurton et al., have reported a clinically relevant method for the generation of CAR T cells with Tscm phenotype using a *Sleeping Beauty* platform. To do so, they incorporated CAR construct with a membrane-tethered IL-15(mbIL15). The authors found that mbIL15 induces survival signals in the mbIL15-CAR T cells via STAT-5 in a CAR signaling-independent manner, without eliciting cell autonomous overgrowth, and compromising antitumor activity. Their data also showed that phenotype of generated long-lived T cells is most similar to Tscm and have a memory-like transcriptional signature (29). Considering the positive effect of cytokines (e.g., IL-15) on persistence and differentiation status of CAR T cells, it is clear that overexpression of cytokines in CAR T cells can help make long-lived CAR T cells.

Interleukin-12 (IL-12) is a heterodimeric molecule composed of p35 and p40 subunits. This cytokine is a key inducer of T cell differentiation toward the Th1 phenotype, while suppressing Th2 development. In the preclinical studies, treatment with recombinant IL-12 has a dramatic antitumor effect against various types of cancer (30–32). Considering antitumor activity of this cytokine, Pegram et al., demonstrated that UCB T cells modified to co-express CD19-specific CAR (1928z) and IL-12 retained a central memory-effector phenotype and showed increased antitumor efficacy *in vitro* and *in vivo* (33).

Many biological effects of IL-12 and IL-18 overlap; however, IL-18 is monomeric, whereas IL-12 is a heterodimeric cytokine. IL-18 was primarily characterized as an inducer of IFN-γ expression in T cells and has been displayed to activate lymphocytes and monocytes without prompting severe dose-limiting toxicity in clinical trials (34). It has been also reported that human recombinant IL-18 (hrIL-18) could significantly improve the engraftment of human CD8^+^ T cells in a xenograft model (35, 36). Thus, IL18 would be a favorable cytokine in boosting the functions CAR T cells in preclinical and clinical studies. Avanzi et al., have developed CAR T cells that constitutively secrete IL-18. Their results revealed that IL-18 not only can enhance CAR T cell survival and antitumor activity both *in vitro* and *in vivo*, but also can activate cells of endogenous immune system in immunocompetent mice (37). In a similar study, Hu et al., discovered that anti-CD19 and anti-mesothelin CAR T cells engineered to secrete IL-18 not only can support *in vivo* engraftment and persistence of CAR T cells but also enhance secretion of IFN-γ and several other cytokines such as IL-2, G-CSF, GM-CSF, TNF-α, IL-17A, and IP-10 (38).

IL-4 is another interesting cytokine with controversial effect on T cell survival. Despite the complex relationship between cancer and endogenous IL-4, administration of supraphysiologic quantities of this cytokine has been extensively explored for the treatment of several cancers. Various *in vivo* studies have frequently revealed potent antitumor activity of exogenous IL-4 in many preclinical models (39–41). Although the efficacy of IL-4 administration in clinical practices has been partial, these studies have led to determination of safe maximum doses in human (42, 43). Taking together, these practices provide a platform for development of additional immunotherapies that include the application or harnessing IL-4. Wilkie et al., have constructed a chimeric cytokine receptor (4αβ) composed of IL-4 receptor α (IL-4Rα) extracellular domain and a shared IL-2/15 βc subunit. Their data revealed that overexpression of this chimeric receptor in anti-MUC1CAR T cells not only results in phosphorylation of STAT3/STAT5/ERK and proliferation, in a similar way to IL-2, but also can kill MUC1-positive tumor cells (44). Although IL-4 receptor is known to signal via janus kinase 1 (JAK 1) and JAK3/STAT6 pathway, in an interesting study, Vella et al., reported that IL-4R inhibits T cell apoptosis in a STAT6-independent manner and suggested that IL-4R in resting T cells may activate a novel signaling pathway to improve T cell survival (45). Overall, although there is no enough data regarding the effect of IL-4 effect on CAR T cell survival *in vitro*, it remains to be determined whether it can improve CAR T cell persistence. In an interesting study, to make CAR T cells resistant against immunosuppressive cytokine IL-4 in a pancreatic cancer model, Mohammed et al., have developed an inverted cytokine receptor in which extracellular domain of IL-4 receptor was fused to an endodomain of IL-7 receptor (4/7 ICR). This approach could enhance antitumor activity of these gene-modified CAR T cells in an IL-4-rich tumor microenvironment (46). Notably, these CAR/ICR T cells were functional in both cytokine and antigen-dependent manner (46). Wang et al., also reported that a novel inverted cytokine receptor IL-4/IL-21 (4/21 ICR) can improve the efficacy of CAR T cell therapy in IL-4-enriched tumor milieu. They showed that, upon binding of IL-4, 4/21 ICR not only activates the STAT3 pathway and polarizes engineered T cells into Th17-like cells but also promotes antitumor cytotoxicity *in vitro*. Moreover, 4/21 ICR CAR T cells could persist and eradicate IL-4-expressing tumors *in vivo* (47).

Altogether, these data indicate that development of strategies for overexpression or repression of some cytokines and/or their cognate receptors in CAR T cells may remarkably enhance the persistence and thereby antitumor activity of these engineered cells. Figure 1 illustrates novel modifications in cytokines and their cognate receptors to enhance CAR T cell persistence for adoptive cancer immunotherapy.

## Pharmacological Inhibitors

Despite the great strides that have been made by CAR T cell therapy so far, some patients still do not respond to this method of therapy due to, for example, paucity or deficiency of long-lived T cells (48, 49). It is well-known that expansion, differentiation, and survival of T cells depend on the integrated signals coming from TCR engagement, cytokine receptors, and costimulatory molecules (50, 51). These signals lead to activation of two main signal transduction networks in T cells, MAPK and PI3K/AKT/mTOR pathways. MAPKs are a widely conserved family of eukaryotic serine/threonine protein kinases. MAPKs regulate various cellular processes such as cell survival, differentiation, proliferation and migration. ERK1/2, JNK1/2 and the p38 are three components of MAPK pathway which have been well characterized (52). The key molecules of PI3K/AKT/mTOR pathway are PI3Ks, AKT, and mTOR. These components have been shown to be frequently hyperactivated in the majority of cancers and have therefore been the focus of many studies in this field. In this regard, various inhibitors for targeting different components of PI3K/AKT/mTOR pathway in cancer cells have been developed. Such studies revealed that inhibition of distal molecules of the MAPK pathway such as ERK by pharmacologic agents or genetic manipulation (gene targeting experiments), significantly weaken the proliferation ability of T cells in mice and humans (53, 54). Considering the role of these agents in generation of less differentiated T cells *in vitro* plus their translational potential for *in vivo* studies, these molecules have nowadays become the favorable targets for improving persistence of CAR T cells. Here we discuss several studies that have tried to enhance CAR T cell survival through manipulation of signaling molecules.

Protein Kinase B (PKB or AKT) pathway, as a key cell survival signal, has prominent role in the multiple cellular functions such as T cells differentiation, survival, and memory formation. Various studies have demonstrated that blocking of AKT signaling pathway in CAR T cells is associated with generation of less differentiated memory cells, thereby conferring survival advantage compared to untreated T cells (50, 55). These data showed that inhibition of Akt signaling during *ex vivo* expansion and priming leads to generation of large numbers of memory CAR T cells with superior antitumor activity (55).

Petersen et al., have demonstrated that antagonism of vasoactive intestinal peptide (VIP) signaling and blockade of PI3Kδ partially block the terminal T cell differentiation during anti-CD28/CD3 bead-mediated expansion. This strategy not only results in enhanced antitumor function of human anti-CD5 CAR T cells but also confers survival advantage compared to untreated anti-CD5 CAR T cells (56). Taken together, these data indicate that synergistic blockade of these signaling pathways is an appealing strategy to improve antitumor activity and persistence of *ex vivo*-expanded anti-CD5 CAR T cells (56). In another interesting study, Perkins et al., have shown that culturing of anti-BCMA CAR T cells with IL-2 and PI3K inhibitor (PI3Ki) leads to an increased frequency of CD62L+ CD8 T cells in the final product. Their data revealed that inhibition of PI3K during *ex vivo* expansion with IL-2 may generate a superior anti-BCMA CAR T cell product for clinical use (57). Likewise, Zheng et al., reported that treatment of CAR T cells with a PI3Ki not only maintains their less differentiation state without affecting their expansion capacity but also improves their persistence *in vivo* which results in reduction of tumor burden (58). However, since PI3k-Akt signaling pathway plays a critical role in mediating survival signals in activated T cells (e.g., through upregulation of Bcl-XL) (59), inhibition of this pathway should be carefully investigated. Moreover, advantage and disadvantage of targeting PI3K-Akt pathway in enhancement of the durability of CAR T cells for immunotherapy through pharmacologic inhibition, gene knocking down/out strategies (e.g., using siRNAs, shRNAs, CRISPR/Cas9, and TALEN techniques) should be thoroughly evaluated.

Mitogen/Extracellular signal regulated Kinase (MEK) is a downstream signaling molecule of MAPK which has a key intermediate role in MAPK pathway. Ebert et al., have demonstrated that MEK inhibition has no profound effect on CD8+ naïve T cell priming in tumor-bearing mice, but essentially increases the abundance of effector-phenotype antigen-specific CD8+ T cells within the tumor. They also showed that MEK inhibition can protect tumor-infiltrating CD8+ T cells from death driven by chronic TCR stimulation while sparing cytotoxic activity. The authors found that combining MEK inhibition with anti-PD-L1 results in synergistic and durable tumor regression. Altogether these data reveal that despite the central importance of the MAPK pathway in some aspects of T cell function, MEK-targeted agents can be compatible with adoptive (CAR) T cell immunotherapy (60).

**Figure 2:**
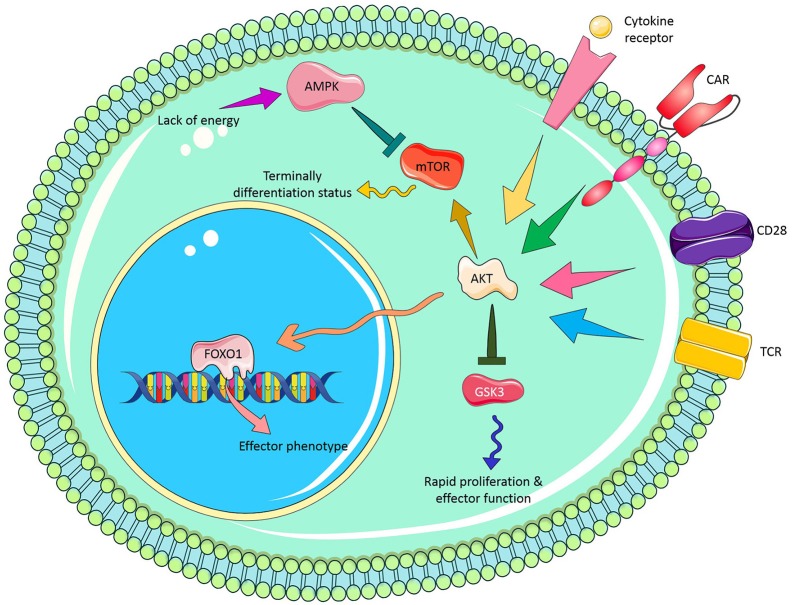
Novel molecular signaling targets to improve CAR T cell persistence.

Glycogen synthase kinase 3 (GSK3) is a constantly active protein in T cells that induces an inactivation signal in naïve T cells. When T cells are activated via CD3z signaling, GSK3 is temporarily repressed following the PI3-kinase/pAKT signaling cascade. This temporal inhibition of GSK3, owing to its phosphorylation, endorses a rapid clonal expansion of newly activated T cell. However, when activated T cell touches peak expansion, GSK3 is rapidly dephosphorylated and activated, resulting in clonal contraction, cytokine loss and activation-induced T cell death. Sengupta et al., showed that pharmacologic inhibition of GSK3 with SB216763 in glioblastoma (GBM)-specific CAR T cells can reduce the expression of Fas ligand (FasL) and exhaustion marker [e.g., programmed cell death protein 1(PD-1)] and increase T cell proliferation resulting in the development of CAR T effector memory phenotype (61). Collectively, these results raise the possibility of inclusion of pharmacological inhibitors such as AKTi, GSK3i. PI3K, and MEKi in successful therapeutic CAR T cell regimens against cancer. Figure 2 illustrates potential molecular signaling targets which can be manipulated for enhancing CAR T cell persistence.

## Conditioning Regimens

In the clinical oncology, during the “countdown period,” usually few days before the CAR T cell therapy, a lymphodepleting conditioning regimen is administered. Lymphodepleting conditioning regimens, comprising IL-2, cyclophosphamide, and fludarabine, are most often used before CAR T cells infusion, allowing for bigger T cell expansion and survival (62–68). During this period, lymphodepleting regimen eradicates homeostatic cytokine sinks, such as IL-7, IL-2, and IL-15, eliminates unwanted immunosuppressive cells, such as regulatory T cells and myeloid-derived suppressor cells and downregulates indoleamine 2,3-dioxygenase (IDO) in tumor cells (67, 69). Furthermore, it induces costimulatory molecules, prevents host anti-CAR immune responses and promotes expansion, function, accessibility, and persistence of adoptively transferred CAR T cells potentially by altering the TME (64, 70). These experiences resulted in the utilization of lymphodepleting conditioning in the CAR T cell clinical trials. Kochenderfer et al., exhibited a correlation between an increase in serum IL-15 levels with clinical response after anti-CD19 CAR T-cell therapy and lymphodepletion (71). Turtle et al., demonstrated that adding fludarabine (Flu) to the lymphodepletion regimen could postpone or abolish host anti-CAR immune responses (which rendered CAR T cell therapy ineffective) and improve CAR T cell expansion and persistence. They observed that proliferation and persistence of CD4+ and CD8+ CAR T cells in the patients who received Flu is remarkably higher than those patients who did not receive Flu. The authors also found that in the patients treated with cyclophosphamide and Flu, pre-conditioning regimens comprised of Flu could improve persistence of CAR T cells and disease-free survival of patients compared to cyclophosphamide alone (72). In aggregate, as a great deal of studies indicate that lymphodepleting conditioning regimens such as cyclophosphamide and fludarabine probably work by a couple of mechanisms besides lymphodepletion, a more suitable term for these approaches might therefore be “conditioning regimen” rather than “lymphodepleting regimen.” It seems that future investigations should further explore the strategies for optimization of conditioning regimens aiming to improve clinical outcomes of CAR T cell therapy.

## Ectopic Expression of Cell Immortalization Genes

Limited replicative lifespan, referred to as replicative senescence, of CAR T cells limits expansion and the long-term persistence of these cells *in vivo*. The replicative senescence also results in the loss of proliferative capability and functional deficit with subsequent physical disappearance and can therefore hinder the long-term therapeutic efficacy of CAR T cell therapy (73). Of numerous factors involved in the controlling of T cell lifespan, telomere is a key factor which is directly related with the T cell senescence (74). It is well-recognized that, similar to the most of human cells, after each cell division, the length of telomere in T cells is shortened. This shortening of telomeres is supposed to be a critical mechanism of cellular senescence following multiple rounds of cell divisions. Recent investigations have suggested that the maintenance of telomere length and replicative ability is associated with the engraftment efficacy and antitumor efficiency of adoptively transferred T cells (75). Rufer et al., have demonstrated that overexpression of human Telomerase Reverse Transcriptase (hTERT) gene in primary human T lymphocytes and CD8+ naïve T lymphocytes can extend their longevity. These transduced T cells express high levels of telomerase and can either maintain or elongate their telomere lengths upon culture for extended periods of time. The authors also showed that transduced T cells not only have a normal 46, XY karyotype and preserve cytotoxic properties, but also represent a negligible apoptosis (73). Bai et al., have transiently transfected anti-CD19 CAR T cells by mRNA encoding TERT to enhance the telomerase activity in these cells aiming to increase proliferation capacity and to delay replicative senescence without risk of immortalization or insertional mutagenesis. Their results showed that compared to conventional anti-CD19 CAR T cells, transfection of mRNA encoding TERT in anti-CD19 CAR T cells leads to improved proliferation and persistence in murine xenograft tumor models of human B-cell lymphomas (76). Collectively, these data demonstrate that transient ectopic expression of TERT would be a new, safe and effective therapeutic approach that improves the persistence of CAR T cells and thereby therapeutic potential of CAR T cells in treating cancers in particular solid tumors. However, safety cautions should be taken particularly in case of stable overexpression of TERT using lentiviral vector transduction.

## Inhibition/modulation of Immune Checkpoints

There are a couple of strategies that can be employed to improve the CAR T cell survival in cancer immunotherapy via targeting immune checkpoints such as CTLA-4, PD-1, LAG-3, and TIM-3. These strategies are including (i) blocking of co-inhibitory molecules such as anti-PD1/PDL1 monoclonal antibodies, (ii) knocking down of co-inhibitory molecules by shRNA expressing vectors, (iii) knocking out of co-inhibitory molecules using gene-editing technologies (e.g., CRISPR/Cas9), changing promoter and site direct mutagenesis, (iv) inhibition of specific transcription factors which are involved in the expression of one or more co-inhibitory /exhaustion molecules. For example, Cherkassky et al., have shown that mesothelin-targeted CAR T cells overexpressing PD-1 Dominant Negative Receptor (PD-1 DNR) which were cotransduced with vectors expressing shRNA against PD-1, had superior antitumor activity compared to unmodified CAR T cells. The authors found that such superior antitumor activity is associated with improved survival of CAR T cells (77). To overcome activation induced cell death (AICD) in anti-GD2 CAR T cells, using anti-PD-1 antibody and siRNA targeting PD-1, Gargett et al., reported that PD-1 blockade/knockdown restores CAR T cell cytokine production and promotes their survival (78). Another study has exhibited that *in vitro* blockage of LAG3 increases the proliferation and production of cytokines and leads to increased formation of memory cells (79). In another interesting study, Chen et al., uncovered nuclear receptor subfamily 4 group a member 1(NR4A1), NR4A2 and NR4A3 as the fundamental transcription factors that trigger dysfunction of T cells. To find the intrinsic drivers of exhaustion, transcriptional signatures between hypofunctional and functional CD8+ T cells has been evaluated. By conducting single cell transcriptomic analysis and chromatin accessibility, these researchers found that transcription factors NR4A1, NR4A2, and NR4A3 are overexpressed and play pivotal roles in driving CD8+ T cell exhaustion in both murine CAR-modified T cells and endogenous lymphocytes as well as their human counterparts which were chronically exposed to tumor or viral infection (80). Considering these results, it may be postulated that targeting these transcription factors may revert CAR T cell exhaustion and enhance their survival. However, this remains to be assessed. In aggregate, it seems that targeting immune checkpoint molecules would be a promising strategy for improving the persistence of CAR T cells. However, as these molecules physiologically maintain immune homeostasis, possible adverse effects such as hyperactivation of immune system and emerging of autoimmune diseases should be emphasized. Table 1 summarizes a list of co-inhibitory molecules which are supposed to be potential candidates for targeting CAR T cells by different means such as genetic engineering, monoclonal antibodies, gene-editing technologies, and gene-knockdown strategies.

**Table 1:** Co-inhibitory molecules which are supposed to be good candidates for targeting CAR T cells.

**Molecule**	**Ligand**	**Receptor contribution**	**References**
CTLA4	CD80 and CD86	Activated T cells	(81)
PD1	PD-L1	Activated T cells, B cells, DCs, NKT cells, Mo1	(82)
Tim-3	Phosphatidylserine, HMGB1, Galectin-9	T cells, B cells, NK cells, NKT cells, DCs, MQ	(83, 84)
LAG-3	MHCII	T cells, B cells, NK cells	(85, 86)
TIGIT	VR, PVRL2, and PVRL3	T cells, NK cells	(87)
The Leukocyte-associated Ig-like Receptor-1(LAIR-1)	Collagen	T cells, B cells, NK cells, DCs, Mo, eosinophils, Basophils, Mast cells	(88)
BTLA	HVEM	T cells, B cells, DCs, Myeloid cells	(89, 90)

## Manipulation of Car Construct

CAR signaling domains apparently play profound roles in the persistence, expansion, and phenotype characteristics of engineered T cells. It has been recognized that 4-1BB and CD28 signaling domains along with CAR constructs not only play a central role in antitumor responses of CAR T cells but also their inclusion in the CAR construct (along with CD3z and as second-generation CARs) could extend T cell survival compared to first-generation CARs (91–93). Furthermore, although both second-generation CARs have shown incredible complete remission rates in patients with B cell malignancies, CD28-based CARs program greater functionality while 4-1BB-based CARs drive higher longevity. The mechanisms for the latter remain to be fully illuminated. Kawalekar et al., have shown that signaling domains of coreceptors CD28 and 4-1BB have remarkable impacts on the metabolic characteristics of CAR T cells. They found that incorporation of 4-1BB in the CAR construct can promote the outgrowth of CD8+ Tcm cells with significantly improved respiratory capacity, enhanced fatty acid oxidation (FAO), and heightened mitochondrial biogenesis. Conversely, CD28-based CAR T cells yielded Tem cells with enhanced glycolytic metabolism (94). Two other studies have also exhibited that CD28-based CARs can induce higher expression level of the anti-apoptotic protein Bcl-XL and higher resistance to AICD when compared to 4-1BB-based CARs (95, 96). Telomerase activity might also contribute to the differences in persistence. One study have revealed that a CD28-based CAR could induce peak levels of telomerase two days after antigen stimulation, which dropped by day 4, while a 4-1BB-based CAR could induce more persistent activity (97). Interestingly, one study have compared clinical outcomes (such as efficacy and adverse events) of two different CD19-tardeted CAR T cells (CD28-based vs. 4-1BB-based CARs) in the patients with B cell non-Hodgkin's lymphoma (B-NHL). Obtained data revealed that both CAR T cells have similar antitumor efficacy, with a complete response (CR) rate of 67% within 3 months for all patients. However, unlike 4-1BB-based CAR T cell therapy which was well-tolerated, severe cytokine release syndrome and neurotoxicity syndrome was observed in the patients received CD28-based CAR T cell therapy (98). The authors suggested that 4-1BB-based CARs have clinical advantage compared to CD28-based CARs for the treatment of B-NHL. These findings, at least in part, highlight how different intracellular signaling domains in the CAR construct can affect on persistence of CAR T cells (94).

Inducible T cell co-stimulator (ICOS) is another costimulatory molecule of the CD28 family that has been shown to be important for optimal immune response. Cytoplasmic domain of this molecule has been frequently included in the CAR construct. Guedan et al., have shown that the presence of ICOS in the CAR structure can significantly increase T cell persistence compared to CD28 molecule. Moreover, it has been exhibited that combining ICOS and 4-1BB intracellular domains in third-generation CARs result in superior antitumor activities and increased persistence *in vivo*. Intriguingly, the location of intracellular domains (the membrane-proximal domains vs. membrane-distal domains) had differential effects in the third-generation CARs. The optimal antitumor and persistence benefits was observed in third-generation CAR T cells with ICOS intracellular domain positioned proximal to the cell membrane and linked to the ICOS transmembrane domain (99). The cytoplasmic tail of ICOS contains an YMFM motif that binds the p85α subunit of class IA PI3K similar to the YMNM motif of CD28. However, compared to CD28, ICOS more strongly activates PI3K (100). Through activation of AKT, PI3K indirectly phosphorylates two negative controllers, tuberous sclerosis complex 2(TSC2) and proline-rich AKT substrate of 40 kDa (PRAS40), which could lead to activation of the mTOR kinase. mTOR kinase is the catalytic subunit of two functionally different complexes, mTORC1 and mTORC2, that coordinately stimulate cell proliferation, cell growth, and survival (101, 102). Therefore, it seems that activation of PI3K/AKT signaling pathway which is downstream of ICOS-based CARs makes ICOS-based CAR T cells more persistent compared to 4-1BB-based CAR T cells indicating their therapeutic potential for clinical practice.

Tumor necrosis factor receptor superfamily, member 7(TNFRSF7or CD27), a member of TNFR/nerve growth factor receptor family, is a homodimeric receptor widely expressed on T cells, NK cells, and B cells (103). While it is not always required for most of T cell responses, upon engagement of CD27 with its cognate ligand (CD70), the activation and survival of CD8+ T cells and their subsequent differentiation into memory cells as well as differentiation of CD4+ T cells into IFNγ-secreting T cells are increased (104–108). Song et al., examined the effect of CD27, as co-stimulatory molecule incorporated in the structure of anti-folate receptor-α (FR) CAR, on proliferation and survival of CAR T cells *in vivo* and *in vitro* (109). Their results showed that, stimulation of FR-targeted CAR T cells in an antigen-dependent manner *in vitro* leads to an increase in the expression of antiapoptotic protein Bcl-XL and makes CAR T cells more resistant to apoptosis (109).

To elucidate the effect of new signaling molecules rather than CD28, 4-1BB and ICOS co-stimulatory molecules in CAR T cells, Foster et al., have activated TLR and CD40 signaling in human T cells using inducible MyD88/CD40 (iMC) via the synthetic dimerizing ligand, rimiducid, to provide potent costimulation to CAR-modified T cells. They found that the concurrent activation of chimeric MyD88/CD40 protein (with rimiducid) and CAR (by antigen recognition) can provide a potent costimulatory signal that enhances T cell survival and boosts T cell proliferation in the context of CAR signaling (110).

It is also well-recognized that optimal signals guiding T cell activation, proliferation, and differentiation require multiple signals, including signal 1 from TCR engagement, signal 2 from co-stimulatory molecules and signal 3 from engagement of cytokine receptors. CAR transgenes which are currently being examined in the preclinical and clinical studies are second-generation CARs that are comprised of a CD3z intracellular domain (TCR signaling) and a costimulatory domain but not an intracellular domain that transduces cytokine receptor signal as a signal 3. Kagoya et al., have constructed a novel CAR transgene which is able to induce signal 3 after antigen recognition and stimulation. This new generation of anti-CD19 CAR construct is comprised of CD3z and CD28 intracellular domains plus a truncated intracellular domain of the IL-2Rβ with a STAT3-binding YXXQ motif (also referred to as 28-ΔIL2RB-z (YXXQ). Obtained data showed that 28-ΔIL2RB-z (YXXQ) anti-CD19 CAR can activate STAT5, STAT3, and JAK kinase signaling pathways in an antigen-dependent manner and, thereby, not only promote proliferation of anti-CD19 CAR T cells but also prevent their terminal differentiation. This new generation of CAR T cells also exhibited superior antitumor effects and higher *in vivo* persistence in different cancer models compared to conventional second-generation CAR T cells that possess only a costimulatory domain such as either 4-1BB or CD28 (111). Altogether, it seems that making more persistent CAR T cells may be achieved by modulation of major intracellular signaling domains (e.g., 4-1BB, CD28, TLRs, or CD40) in CAR T cells. However, owing to the central importance of these signaling molecules in different cellular functions, it is necessary to fully characterize the mechanisms that govern CAR T cell survival.

## Knocking Down of Proapoptotic Molecules and Overexpression of Antiapoptotic Proteins

Through maintaining the homeostatic balance between cell proliferation and cell death, apoptosis regulates cell fate, and survival (112). Apoptosis can by mediated by two ways including death receptor-independent and dependent pathways, which are linked to the release of cytochrome C from the mitochondria (113). The release of cytochrome C is a crucial point in the switching on/off of the apoptosis process and is controlled by interaction between proapoptotic and antiapoptotic proteins, for example, members of the Bcl-2 family, inhibitor of apoptosis (IAP) proteins (e.g., survivin, Akt, and heat shock proteins) and other proteins like cellular FLICE-inhibitory protein (cFLIP) (114–116).

Brink et al., have shown that upregulation of proapoptotic proteins such as Bim, Bid, FasL is associated with progressive T cell differentiation and loss of self-renewal capacity. They have also found that increased cumulative T cell signaling in the alloreactive m1928z T cells [in response to CD19 and alloantigens] not only can cause upregulation of Bim, Bid, FasL but also leads to their chronic activation and thereby functional exhaustion and apoptosis (117). Although many antitumor agents have been used to target the proteins involved in apoptosis to induce cancer cell death or enhance the sensitivity of cancer cells to certain cytotoxic drugs or radiation, targeting of proapoptotic proteins have not been considered enough in CAR T cell therapy to make more persistent CAR T cells. There are many investigations underscoring that targeting proapoptotic protein in T cells can improve T cell longevity (118–124). Thus, it seems that knocking down or knocking out of proapoptotic proteins such as Bim and/or Bid, by shRNA and siRNA or CRISPR/Cas9 respectively can lead to increased survival of CAR T cells in a similar way to tumor cells which acquire resistance to apoptosis through downregulation or mutation of proapoptotic proteins. However, possible adverse effects (e.g., emerging autoimmune diseases) and antitumor ability of CAR T cells due to pharmacologic and/or genetic inhibition should be assessed in the future experiments. It should be also noted that the caution should be warranted as these findings which are related to unmanipulated T cells may not apply to CAR T cells. In aggregate, it looks like that manipulation of proapoptotic genes could improve T cell survival and would be beneficial in increasing CAR T cell persistence and, in turn, improving their antitumor effect. Table 2 represents potential proapoptotic molecule targets for enhancing CAR T cell persistence.

**Table 2:** Potential proapoptotic molecule targets for enhancing CAR T cell persistence.

**Molecule**	**Function**	**References**
Bad	Promoting apoptosis	(125)
Bax	Promoting apoptosis	(125)
Bak	Promoting apoptosis	(125)
Bid	Promoting apoptosis	(117, 118, 120)
Bim	Promoting apoptosis	(117–120, 122, 123)
Nip3	Promoting apoptosis	(124)
Nix	Promoting apoptosis	(124)
Siva	Promoting apoptosis	(126)

Antiapoptotic proteins such as some of Bcl-2 family members [e.g., Bcl-2, Bcl-XL, Bcl-W, myeloid cell leukemia 1(MCL-1), and A1] act as cell survival regulators and are involved in a wide range of cellular activities in both physiological and pathological conditions (127, 128). The main function of the these antiapoptotic proteins is to prevent the release of apoptosis induced factor(AIF) and cytochrome C into cytoplasm as these proteins directly activate the caspases (127). These proteins also bind to proapoptotic proteins such as Bid, Bax and Bad which target mitochondrial membrane integrity (125). Although targeting the expression or activity of these antiapoptotic factors is a therapeutic strategy in cancer therapy, in some therapeutic modalities such as adoptive T cell therapy, it is required to make T cells more resistant to apoptosis in a hostile tumor microenvironment aiming to enhance their survival and, thereby, the efficacy of ACT. Several studies have shown that overexpression of Bcl-2 family members in immune cells such as T cells leads to superior cell survival (129, 130). For example, Charo et al., have demonstrated that overexpression of Bcl-2 in TILs, even after deprivation of IL-2, can increase T cell survival and make these cell more resistant to cell death (129). Antiapoptotic protein survivin is a member of IAP family which directly inhibits the activity of caspases 3 and 7 (131). Song et al., have demonstrated that survivin expression is not only sufficient to restore proliferation and antagonize apoptosis in costimulation-deficient T cells but also can rescue T cell expansion *in vivo* (132). Although there has not been any study that overexpressed these antiapoptotic proteins in the CAR T cells, it is expected that overexpression of antiapoptotic proteins such as members of Bcl-2 family in CAR T cells make these cells more resistant to proapoptotic signals which are abundant in a hostile TME. However, this yet remains to be assessed. Similar to knocking down/out of proapoptotic proteins, the possible adverse effects and cautions associated with overexpression of antiapoptotic proteins in CAR T cells should be also addressed.

## Manipulation of Redox Regulatory System

The balance between the production and removal of reactive oxygen/nitrogen species (ROS/RNS) is called redox balance and it is vital for physiological activity of the cells (133). It is important to realize that redox-based molecules are critical mediators of key functions in physiological systems and are essential to immunity against diseases. Redox balance is tightly regulated through multiple complex systems such as thioredoxin (Trx) system, glutathione system, cytochrome P450 system, superoxide dismutase, catalase, and peroxiredoxins. However, if this balance breaks down oxidative stress increases, leading to irreversible damages to cell survival (134, 135). It has been well-documented that ROS can seriously hamper the efficacy of active immunotherapy and adoptive transfer of T cells into patients (136–139). For example, increasing ROS in tumor infiltrating T cells has been also associated with overexpression of PGE2 and reduction of antioxidant enzymes such catalase, Cu/Zn, and Mn-SOD (136). Ando et al., have exhibited that enhanced expression of antioxidant enzyme catalase in human T cells can protect them against reactive oxygen species. The authors showed that catalase transduction makes CD4+ T cells less sensitive to H2O2-induced loss-of-function. These genetically-modified T cells are also more resistant to oxidative stress-induced cell death after coculture with activated granulocytes or exposure to the oxidized lipid 4-hydroxynonenal, or H2O2 (140). In another interesting study, Maarten et al., have demonstrated that T cells engineered to coexpress CAR and catalase (CAR-CAT) perform superior compared to CAR-expressing T cells. They also found that CAR-CAT T cells express high levels of intracellular catalase and have a reduced oxidative state with less ROS accumulation in both the basal and activation states while preserving their antitumor activity even in the presence of high H2O2 concentrations. Furthermore, T cells coexpressing CAR and catalase exert a significant bystander protection of untransduced effector cells even in the presence of high H2O2 concentrations (141). It has been shown that modulation of various signaling molecules [e.g., NF-κB and P38α] and pathways [e.g., mTOR/ p70S6 Kinase (p70S6K] through inducing antioxidant status makes T cells more resistant to ROS-mediated cell death (136, 142). Furthermore, many studies have shown that the antioxidant agents such as bioflavonoids (Theaflavins), curcumin and its derivatives (e.g., curcuminoids and curcumin diferuloylmethane) influence on T cell survival by different means like inhibition of COX-2 and restoring the activity of NF-κB (136, 138, 143, 144). Therefore, it seems that modulation of antioxidant proteins (e.g., catalase, NF-κB, and P38α) and/or joint application of antioxidant agents with CAR T cells can extend the longevity of the cells through making them more resistant to oxidative stress-mediated apoptosis. It should be also noted that the caution should be warranted as these findings which are related to unmanipulated T cells may not apply to CAR T cells.

## Blunting Host Immune Responses

The development of anti-CAR immune responses which rendered CAR T cell therapy ineffective has been described by some studies. For instance, in a recent clinical trial conducted by turtle et al., they have demonstrated that development of CD8+ T cell-mediated anti-CAR immune responses following CAR T cell administration in some patients leads to poor CAR T cell persistence and increased risk of relapse (64). In another clinical trial, the development of anti-CAR immune responses led to the development of anaphylaxis in a patient after receiving several individual doses of anti-mesothelin CAR T cells derived from a murine anti-human mesothelin scFv (145). Jensen et al., have also demonstrated that anti-transgene rejection responses can contribute to poor persistence of adoptively transferred CD20/CD19-specific CAR T cells (146).

To overcome host anti-CAR immune responses and potentially reduce the immunogenicity of CARs, two strategies has been so far introduced: (i) using fully human sequences in CAR constructs instead of murine sequences, (ii) simplifying the CAR structure and reducing the size of the CAR's antigen-binding domain. In a clinical trial, Brudno et al., have assessed the safety, feasibility, anti-lymphoma activity and immunogenicity of a second-generation fully human anti-CD19 CAR T cells (Hu19-CD828Z) in 20 patients with B-cell lymphoma. Their data showed that there was no host anti-CAR response in the treated patients. In another interesting study, to bypass potential anti-CAR immunogenicity, Lam et al., simplify the structure of the CAR's antigen-binding domain by using a fully human heavy-chain-only binding domain without a light-chain domain (also designated as FHVH33-CD8BBZ). In contrast to a scFv, this fully human heavy-chain-only antigen-binding domain has neither linker nor light chain which are potentially immunogenic components of scFv in the CAR constructs. In addition, the smaller size of this CAR is another advantage which may greatly reduce anti-CAR immune response (147). Obtained data revealed that, similar to conventional CAR T cells, after exposure to B-cell maturation antigen (BCMA) positive tumor cells, T cells expressing FHVH33-CD8BBZ can lyse tumor cells, produce cytokine and eradicate tumor cells *in vivo* (148).

Altogether, these findings indicate that primary barrier to therapeutic efficacy of CAR T cells is their poor persistence. This barrier not only provides the rationale to prospectively modify clinical trial protocols (e.g., by avoiding constant exposure to murine CAR constructs) and/or CAR constructs (e.g., using humanized/fully human-derived CARs or simpler/smaller CARs) aiming to improve CAR T cell persistence but also alleviate the possibly related toxicity.

## T Cell Subset Selection

In spite of effective clinical outcomes which have been achieved with the CD19 CAR T cell therapy in B-ALL, some patients have not responded to therapy or relapsed. The basis for this difference in therapy outcome has not been fully understood. One of the reasons can be that T cell qualities (e.g., cell persistence and functionality) are not necessarily the same between different T cell subsets and among T cells from different patients. Two most important subsets of T cell in immunotherapies are memory (include stem cell memory, central memory and effector memory) and naïve T cells which are currently used for formulation of CAR T cell products of uniform composition (149, 150). It is interesting that naïve and memory cells in CD4+ and CD8+ have different properties in terms of cellular kinetic, longevity and function (149, 151, 152). There was no report regarding T cell subset isolation in the CAR T cell-based studies before 2016 (149). However, in 2016, Sommermeyer et al., for the first time, isolated CD4+/CD8+ Tn(T naïve), Tcm, and Tem from patients with B cell malignancies and normal donors to generate anti-CD19 CAR T cells (153). Adoptive transfer of CAR T cells generated from different T cell subsets revealed that, CAR T cells derived from CD4+ naïve(Tn) and CD4+ memory (Tm) T cells have greater antitumor activity and higher cellular persistency in the blood 10 days after infusion. Also, CD8+ CAR T cells from Tcm showed superior survival and greater proliferation capacity *in vivo* compared to CD8+ Tn- and CD8+Tem-derived CAR T cells. These data collectively indicate that CAR T cell formulation based on distinct T cell composition can provide uniform potency compared to formulations derived from bulk T cells that are composed of phenotypically heterogeneous cell subsets (153). By large-scale production of clinical-grade CD19-redirected CAR Tscm cells, Sabatino et al., reported that CAR–modified CD8+ Tscm cells mediate robust and long-lasting antitumor responses against systemic ALL xenografts and exhibit improved metabolic fitness compared to conventional CD8+ CAR T cells generated with standard clinical protocols (150). In a comprehensive study, Turtle et al., have demonstrated that cell product composition [CD8+ Tcm vs. bulk CD8+ T cells], cell dose [dose level 2 (2 ×106 CAR+ T cells/kg vs. dose level 1 (2 ×105 CAR+ T cells/kg)], tumor burden [higher percentage of blasts in the bone marrow (BM) vs. lower percentage of blasts in the BM], and the conditioning regimen [Cy/Flu vs. Cy or Cy/etoposide) could contribute to *in vivo* persistence and expansion of CAR T cells (64). Pule et al., have engineered Epstein-Barr virus (EBV)-specific CTLs to express a CAR directed to the diasialoganglioside GD2-an antigen expressed by human neuroblastoma. They reasoned that these anti-GD2CAR-expressing T cells would receive optimal costimulatory signals following engagement of their native receptors while signaling through chimeric receptors would improve their survival and antitumor activity. Their data proved that EBV-specific CTLs encoding an anti-GD2CAR in subjects with neuroblastoma can indeed extend the survival longer than anti-GD2CAR T cells lacking virus specificity (154).

By considering the kinetic, longevity and persistence of different T cell subsets (149, 151, 152, 155) and the variation in different portions of T cell subsets in patients (64, 153, 156), it seems that having a standard and uniform protocol with the same outcome in different patients cannot be achieved unless selection of T cell subsets before modification of T cells for CAR T cell generation or other T cell-based therapies is considered. Although above studies showed that naïve and central memory T cells in the defined CD4+:CD8+ composition has higher persistence and superior antitumor activity, the cost of T cell selection procedure, achieving a standard and uniform protocol for selection of special T cell subsets in sufficient number are some challenges ahead which should be fully addressed (149). However, some preclinical and clinical data supports the potential benefits of selecting specific T cell subsets for CAR T cell therapy for improving the efficacy and reproducibility of cancer immunotherapy.

## Concluding Remarks

**Figure 3:**
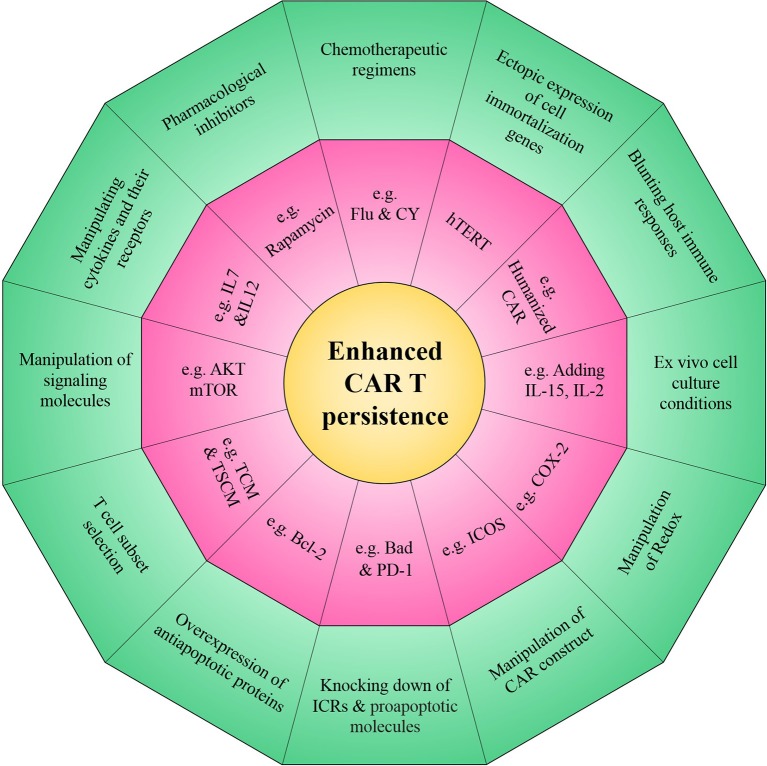
Novel strategies to enhance the persistence of CAR T cells. The overall aim of these approaches is to improve intrinsic CAR T cell fitness and allow to elicit optimal CAR T cell persistence in the setting of many intrinsic and/or extrinsic barriers operative within a harsh tumor microenvironment.

The primary barrier to therapeutic efficacy of CAR T cells is limited persistence and it therefore provides the rationale to prospectively program CAR T cells for longer survival following adoptive transfer. In this review, we comprehensively discuss several strategies which have been established to improve the persistence of CAR T cells (such as introducing novel costimulatory domains into CAR T cells, utilizing various cytokine recipes and pharmacological interventions in the *ex vivo* culture of CAR T cells) (Summarized in Figure 3). It is anticipated that new generations of optimized CAR T cells could improve antitumor immunity so that the combinatorial application of other immunotherapeutic modalities with CAR T cells may synergistically amplify the persistence of CAR T cells. However, it should be noted that concerns regarding emerging lymphoproliferative diseases, in particular for overexpression of antiapoptotic genes and/or knocking down/out of proapoptotic genes, and cytokine induced toxicity should be assessed. It seems that better understanding of the various features of tumor and its surrounding microenvironment that are problematic for persistence of CAR T cells will help us develop new generations of CAR T cells that are more potent in overcoming poor persistence.

## Author Contributions

HM and JH conceived the review. LJ, EM, and KF-M undertook the initial research. All authors were involved in writing and reviewing the manuscript, and all authors contributed to the final version.

## Conflict of Interest

The authors declare that the research was conducted in the absence of any commercial or financial relationships that could be construed as a potential conflict of interest.

## Abbreviations

IAPs: Inhibitors of Apoptosis Proteins
HSP: Heat-Shock Proteins
FasL: Fas Ligand
hBcl-2: Human B-Cell Lymphoma 2
Bcl-xL: B-Cell Lymphoma-Extra Large
rhIL-2: Recombinant Human Interleukin-2
KO: Knock Out
AICD: Activation Induced Cell Death
Nip3: Nineteen Kd Interacting Protein-3
Nix: Nip-Like Protein X
TNFR: Tumor Necrosis Factor Receptor
NB: Neuroblastoma NB
mbIL15: Membrane-Bound Chimeric IL-15
UCB: Umbilical Cord Blood
ALL: Acute Lymphoblastic Leukemia
JAK: Janus Kinase
STAT: Signal Transducers and Activators of Transcription
TGF-β: Tumor Growth Factor- β
MAPK: Mitogen-Activated Protein Kinase
mTOR: Mammalian Target of Rapamycin
PI3K: Phosphoinositide 3-Kinases
PSMA: Prostate-Specific Membrane Antigen
VIP: Vasoactive Intestinal Peptide
iMC: Inducible MyD88/CD40
MEK: Mitogen/Extracellular Signal Regulated Kinase
Tn: T naïve
Tcm: T central memory
Tem: T effector memory cells
PBMC: Peripheral Mononuclear Cell
BM: Bone Marrow
EBV: Epstein-Barr virus
AIF: Apoptosis Induced Factor
IL-2Rβ: IL-2 Receptor β-Chain
CTLA-4: Cytotoxic T-Lymphocyte-Associated Protein4
PD-1: Programmed Cell Death Protein 1
Tim3: T cell Immunoglobulin and Mucin Domain
LAG3: Lymphocyte Activation Gene 3
TIGIT: T cell Immunoreceptor with Ig and ITIM Domains
NFAT: Nuclear Factor of Activated T-cells
ROS: Reactive Oxygen Species
AMPK: AMP-activated Protein Kinase
mTOR: Mammalian Target Of Rapamycin
PGC-1α: PPAR-Gamma Coactivator 1α
BTLA: B- and T-Lymphocyte Attenuator
HVEM: Herpes Virus Entry Mediator
NR4A1: Nuclear Receptor subfamily 4 group A member 1
SHP-1: Src (Sarcoma) Homology 2 Domain Phosphatase-1
SHP-2: Src (Sarcoma) Homology 2 Domain Phosphatase-2
BATF: Basic Leucine Zipper ATF-Like Transcription Factor
SKP2: S-phase Kinase-associated Protein 2
LAIR-1: The Leukocyte-associated Ig-like Receptor-1
ERK1/2: extracellular signal–regulated kinase1/2, AKT, Protein Kinase B
TSC2: Tuberous Sclerosis Complex 2
PRAS40: The Proline-Rich Akt Substrate of 40 kDa
mTORC2: mammalian target of rapamycin complex 2
TNFRSF7: Tumor Necrosis Factor Receptor Superfamily Member 7
MCL-1: Myeloid Cell Leukemia 1
Trx: Thioredoxin
HIF1-α: Hypoxia-Inducible Factor 1 –alpha
AP-1: Activating Protein-1
TAM: Tumor-Associated Macrophages
NO: Nitric Oxide
COX-2: Cyclooxygenase-2
MAPK: Mitogen-Activated Protein Kinase
p70S6K: p70S6 Kinase
ROS: Reactive Oxygen Species
PD-1 DNR: PD-1 Dominant Negative Receptor
TME: Tumor Microenvironment
PD-1 DNR: PD-1 Dominant Negative Receptor
BTLA: B- and T-lymphocyte attenuator
FAO: Fatty Acid Oxidation
ICOS: Inducible T cell Co-Stimulator
ROS/RNS: Reactive Oxygen/Nitrogen Species
NF-?b: Nuclear Factor-κB
FOXO1: Forkhead Box Protein O1
Treg: Regulatory T cell
MDSC: Myeloid Derived Suppressor Cell
iNOS: Inducible Nitric Oxide Synthase
hTERT: Human Telomerase Reverse Transcriptase
5-FU: 5-fluorouracil.
